# Direction and distance dependency of reaching movements of lower limb

**DOI:** 10.1371/journal.pone.0290745

**Published:** 2023-08-25

**Authors:** Kyosuke Oku, Shinsuke Tanaka, Noriyuki Kida

**Affiliations:** 1 Graduate School of Human and Environmental Studies, Kyoto University, Kyoto, Japan; 2 Faculty of Arts and Sciences, Kyoto Institute of Technology, Kyoto, Japan; 3 Institute for Liberal Arts and Sciences, Kyoto University, Kyoto, Japan; Kennedy Krieger Institute/Johns Hopkins University School of Medicine, UNITED STATES

## Abstract

Efficient body movement is required in our daily lives, as it facilitates responding to the external environment and producing movements in various directions and distances. While numerous studies have reported on goal-directed movements in the frontal direction during gait initiation, there is limited research on the efficient movement of the lower limbs in multiple directions and distances. Therefore, we aimed to examine changes in the kinematics of lower-limb reaching movements to determine skilled motor ability in terms of direction and distance. Sixteen adults (10 male participants) were requested to reach targets projected on the floor in seven directions and at three distances for a total of 21 points. The reaching time slowed down for the contralateral side (right foot to left-sided target) and was caused by a slower start of the toe movement. To identify the cause of this delay, we analyzed the onset of movement at each joint and found that movement to the contralateral side starts from the hip, followed by the knee, and subsequently the toe. The time-to-peak velocity was also calculated, and the motion required to reach the target in the shortest time varied depending on direction and distance. These results suggested that movement kinematics vary with direction and distance, resulting in a slower reaching time on the contralateral side. The results of our study hold promise for potential applications in sports and rehabilitation.

## Introduction

We can perform efficient movements while adapting well to the external environment in our daily lives. To ensure appropriate movement output, various factors should be considered, including the surrounding environment and an individual’s physical condition. For example, it is necessary to change the direction and calculate the target distance when moving. Efficient reaching movements in the upper limb for direction and distance have been investigated [1, 2]. In particular, the peak velocity of the fingertips and the reaching time depend on the direction of movement [3]. Furthermore, different strategies that can facilitate reaching the target fastest are used [4]. These actions are thought to involve the brain optimizing its control of the body to move faster and more efficiently [5]. However, this change in reaching movement kinematics with regard to direction and distance is limited to the upper limb. Reaching with the lower limbs is expected to have different characteristics from reaching with the upper limbs because it involves the additional function of supporting the body while moving by manipulating the legs. In sports, such as soccer, it is necessary to reach with the lower limbs quickly and accurately. Thus, accurately moving the lower limbs within a limited time and space, can directly affect performance in sports. Therefore, understanding how to perform effective movements while supporting the body can lead to a more detailed understanding of whole-body movements.

Regarding the reaching movement of the lower limb, much is still unknown about movements in various directions and distances. Walking is a goal-directed movement that involves the use of the lower limbs. It is believed that during walking, stride length and step frequency are adjusted according to the goal of walking at a specific speed [6]. Gait initiation among walking movements is the study of the process from a stationary state to the first step of walking. Studies on gait initiation have reported that electromyography (EMG) of the ankle muscles that control the posture for lifting the legs off the ground is correlated with walking speed in the anterior direction [7]. Furthermore, studies on the center of pressure (CoP) shift have reported that the transition of the CoP before the initiation of walking is related to walking speed in the anterior direction [8]. Many studies on lower-limb movements have focused on movements in the anterior direction [9, 10]. In a study that investigated the initiation of walking in directions other than the anterior direction, the time required for the heel to be lifted off the ground was reportedly delayed when walking to the opposite side at 15° from the anterior direction (walking to the left with the right foot), and that the lateral CoP shift before the heel was lifted off the ground was larger on the contralateral side than on the ipsilateral side [11]. However, the range of investigation is limited (four directions: three on the same side and one on the opposite side), and it is necessary to consider movements in a wider range of directions. In addition, when considering actual movement situations, it is necessary to consider movements at various distances in addition to directions to increased spatial resolution. Therefore, in this study, we aimed to investigate effective movements over a wide range by focusing on the multidirectional and multidistance movements of the lower limbs.

Several studies on gait initiation using the lower limbs have employed electromyography (EMG) or the CoP as variables; however, studies that utilize three-dimensional (3D) data are limited. In studies on gait initiation, some have measured the EMG of the ankle as a posture control for lifting the leg off the ground [7], while studies examining CoPs have utilized force plates [8]. However, 3D data are considered useful for understanding walking movements, particularly in hip kinematics, which has been suggested to have a significant contribution to the work performed in the forward direction [12]. In lower limb movements, it is believed that the work distribution varies among joints and that these characteristics change with alterations in movement direction and distance. Therefore, in this study, we aimed to investigate the effective reaching movements of the lower limbs using 3D data.

In this study, based on previous research, we hypothesized that the time to reach the opposite side would be delayed in lower-limb reaching movements and proceeded to investigate 3D motion data. If the time to reach varies depending on the direction, we aimed to examine the preparatory movements that create this difference and the speed strategies that vary by direction and distance to achieve faster reaching. These hypothesis tests were conducted to elucidate more effective movements in a wide range of multidirectional and multidistance lower-limb movements. Our findings have potential applications in sports and rehabilitation.

## Methods

### Participants

Sixteen healthy adults (10 male and 6 female participants), aged 23.00 ± 2.55 years (mean ± SD), participated in this study. All participants were right-footed, as assessed using the Waterloo Footedness Questionnaire [13]. Participants were selected based on the criteria that they had normal vision and no experience playing soccer at a competitive level. The reason for excluding persons with soccer experience was that we wanted to examine general human function, not that of those who were skilled in leg manipulation. We selected a sample size (n = 16) based on previous studies [14–19]. Prior to the experiments, the purpose of the study and the procedure were explained to the participants, and written informed consent was obtained from each participant. This study was approved by the Ethics Committee of the Graduate School of Human and Environmental Studies, Kyoto University, and was conducted in accordance with the principles of the Declaration of Helsinki.

### Experimental setup and apparatus

**Fig 1:**
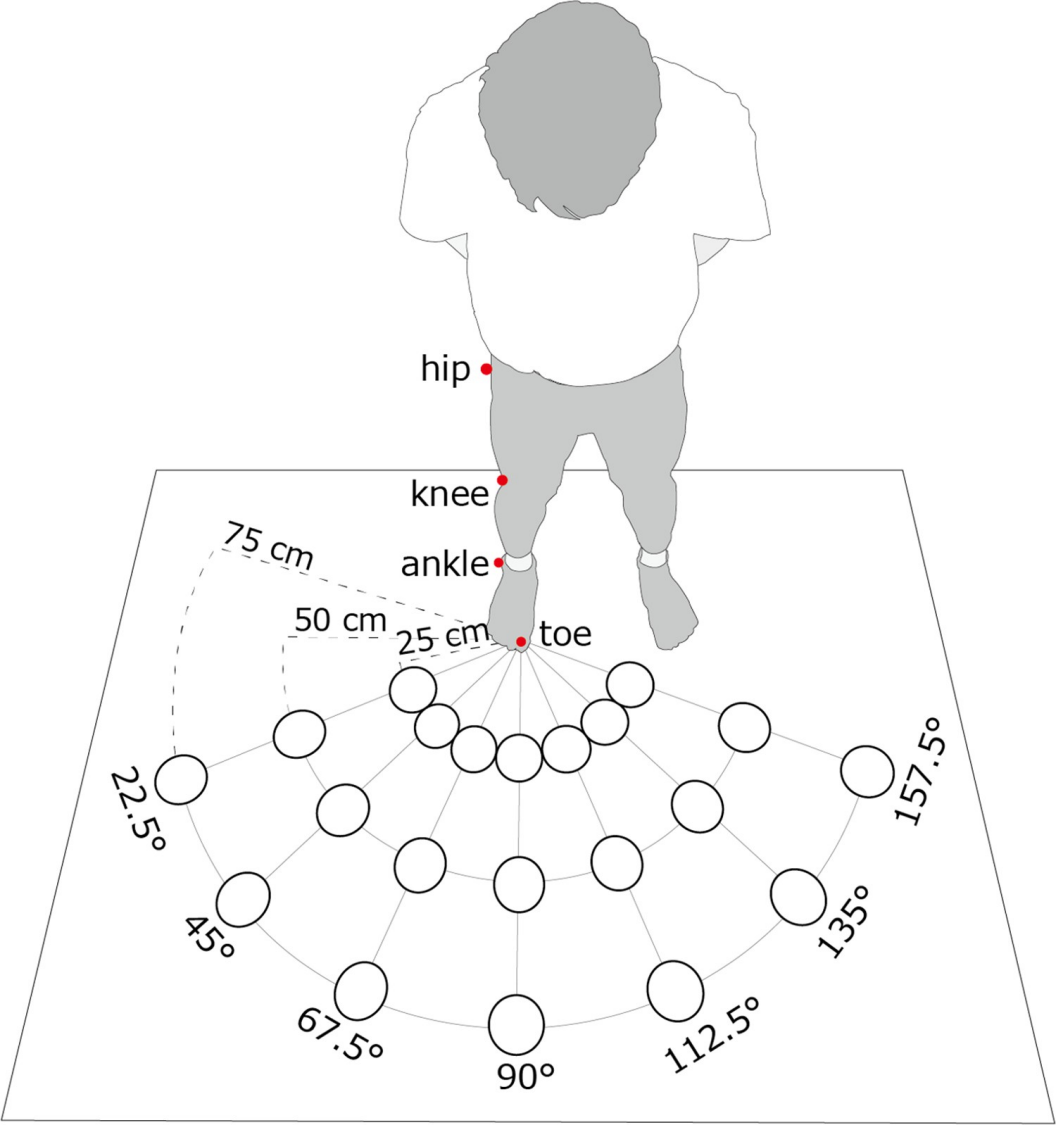
Overview of the experimental setup. It depicts the distances and directions of the projected visual stimuli and the location of the infrared markers.

A schematic of the experiment is presented in Fig 1. The participants were instructed to stand upright on a wooden platform (180 cm wide and 90 cm deep) placed in a dim room. A projector (H6530BD, Acer) placed approximately 200 cm above and 180 cm in front of the participant’s right foot projected the visual targets onto the floor. The visual targets were white circles with a diameter of 10 cm that were drawn using custom software written in Visual Basic 2017 (Microsoft Visual Studio, Microsoft) and run on Windows 10 via VGA outputs. A total of 21 visual targets in seven directions (22.5°, 45.0°, 67.5°, 90.0°, 112.5°, 135.0°, and 157.5°) and at three distances (25.0, 50.0, and 75.0 cm) from the great toe of the right foot were used. Regarding the number of movement directions, we set a larger number of directions than in previous studies and determined the maximum number based on pilot experiments so that participants would not experience performance decline due to fatigue [4, 11]. The farthest distance from the visual target was set based on the approximate criterion that normal adults could reach with a single movement [20–22].

Kinematic data were obtained using a motion-capture system (OptiTrack, NeuralPoint). Reflective markers of 4 mm diameter were placed on the tip of the right second toe, while those with a diameter of 16 mm were placed on the surface of the right ankle (lateral malleolus), knee (lateral femoral condyle), and hip (greater trochanter). The spatial locations of the four reflective markers were captured using six infrared cameras (OptiTrack Flex 3) with a temporal resolution of 100 Hz. A software-controlled infrared LED (tip diameter of 5 mm and wavelength of 850 nm) was placed on the floor and synchronously illuminated with the visual targets to enable the detection of the visual stimulation onset using the motion-capture system.

### Procedure

First, the participants performed a motor task in which they reached the target with their right foot. The participants stood upright with their heels on the platform and stabilized their posture before starting the task. One of the 21 visual targets (seven directions and three distances) was presented to the participant with a 1–4 s random delay after using a beep to cue the onset. As soon as the participant visually perceived the target, he/she tried to reach it with his/her right foot as per the following instruction: “reach for the target with the toe as quickly and accurately as possible.” The participants completely lifted their feet, and upon reaching the target, the participants were asked to keep the toe on the target for 2 s to verify the termination of one trial. Trials were consecutively conducted for all 21 locations at 5-s interval. Subsequently, this set of 21 trials was repeated four times (84 trials in total), with a 2-min interval to prevent fatigue. For clearly failed trials, the participants were asked to perform the movement again under the same conditions during the experiment. The participants practiced the reaching task 10 times prior to the actual session.

### Data analysis

The time series data of the three-dimensional location of the reflective markers were preprocessed using a second-order Butterworth low-pass filter with a cutoff frequency of 10 Hz [23]. The positional data were differentiated in time by 3-point differential algorism to obtain the velocity at each moment for each point. The velocity in three axes in each frame for each point was synthesized to induce instantaneous velocity in 3-dimentional space. The onset and offset of the movement were defined by each criterion at each point. For the toe, the first of the five consecutive frames in which the velocity exceeded 30 cm/s was defined as the onset, whereas the last of the five consecutive frames in which the velocity fell below 30 cm/s was defined as the offset of the movement. This threshold was set to 10% of the speed of the fastest movement among the 21 points on the average of all participants. In the same manner, the criteria for onset and offset for each point were set as 28.0, 18.0, 9.6 cm/s for ankle, knee, and hip, respectively.

**Fig 2:**
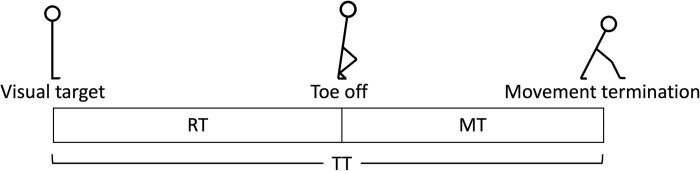
Representative variables arranged in a time series. RT (Reaction Time) represents the time from the presentation of a visual target to the start of the toe movement, MT (Movement Time) represents the time from the start of the toe movement to the end of movement, and TT (Total Time) represents the time from the presentation of a visual stimulus to the end of movement (RT plus MT).

To evaluate the kinematics of the lower limb, we calculated the following indices and present the representative variables in Fig 2: reaction time (RT), was defined as the duration from onset of target presentation to that of movement of the toe; movement initiation for each point (ankle, knee, and hip), was defined as the duration from onset of target presentation to that of movement; movement time (MT), was defined as the duration from onset to termination of toe movement; total time (TT), was defined as the sum of RT and MT, indicating the duration from onset of target presentation to termination of toe movement; and time-to-peak velocity, was defined as the duration from onset of target presentation to the appearance of maximum velocity for each point (toe, ankle, knee, and hip).

### Statistical analysis

We conducted a two-factor analysis of variance of direction and distance for TT, RT, and MT to examine how movement changes with direction and distance (3×7 analysis of variance [ANOVA]). If a simple main effect was detected, multiple Bonferroni-corrected comparisons were performed. The movement initiation at each point was analyzed using a three-factor ANOVA (3×7×4 ANOVA): distance, direction, and point (toe, ankle, knee, and hip). If a two-way interaction effect was detected, it was applied to a two-factor analysis of variance of direction and point for each of the three distances (a 7×4 ANOVA was performed three times). If a single interaction effect was detected, multiple Bonferroni-corrected comparisons were performed to examine the differences in movement initiation between points at each distance. For the time-to-peak velocity of each point, a two-factor analysis of variance of direction and distance was also conducted (7×3 ANOVA was performed four times). If a single interaction effect or main effect was detected, multiple Bonferroni-corrected comparisons were performed to examine how the time-to-peak velocity differed with distance and direction for each point.

## Result

**Fig 3:**
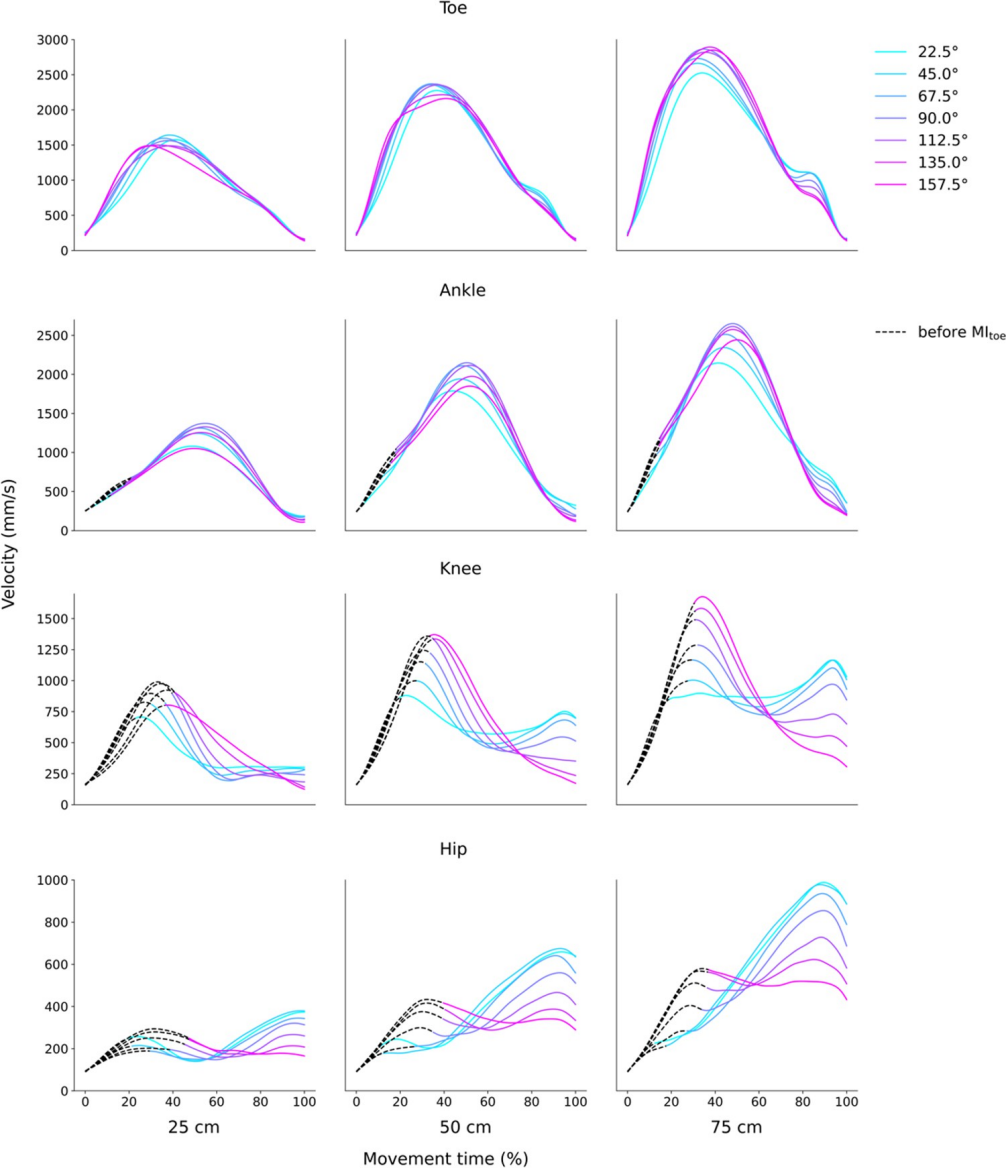
Velocity profile of each joint in relation to distance. The dotted line shows the change in velocity before the initiation of toe movement.

The velocity profile from initiation to completion is shown in Fig 3. The velocity profiles of the toe and ankle were largely similar, depicting a single peak that increased with the distance moved. However, there were two peaks in the knee and hip as MT increased: a large peak preceded a small peak in the knee, while the small peak preceded the large peak in the hip. In both cases, the trend became more prominent as the distance increased.

**Fig 4:**
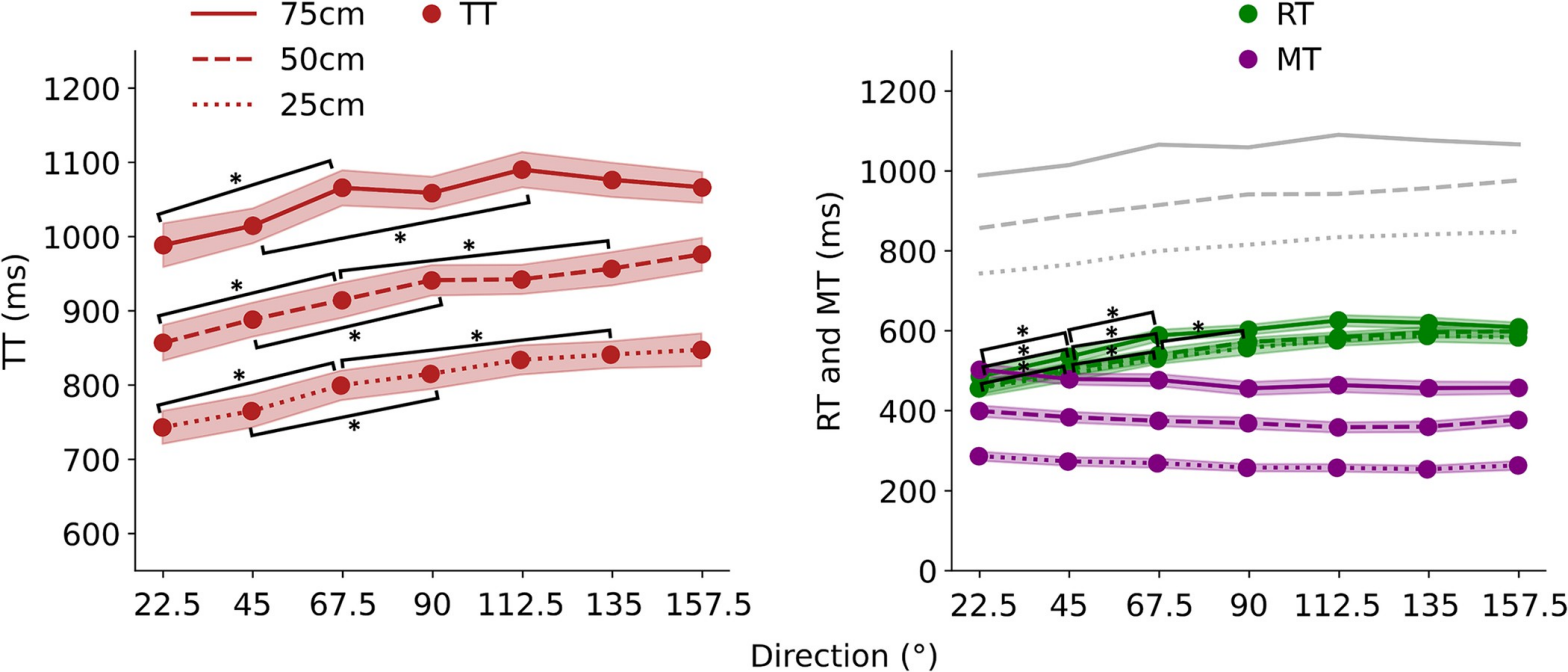
TT and RT, MT. Left panel: TT is the duration from visual stimulus presentation to movement termination. Right panel: RT is the duration from visual stimulus presentation to the movement initiation of the toe; it represents the movement initiation in this study. MT is the duration from movement initiation to completion. The lightly colored bands in both panels indicate SE (Standard error). The significant difference between each joint at 0.5% level is shown. TT is shown in gray in the right panel for reference. TT, total time; RT, reaction time; MT, movement time.

The average values of total time (TT), RT, and movement time (MT) are shown in Fig 4. TT increased with distance and direction angle, particularly up to a reaching distance of 50 cm (Fig 4. left panel). To determine more details about body movements, we divided TT into RT and MT; the average values are shown in Fig 4 (right panel). MT tended to decrease with an increase in direction angle, whereas RT appeared to increase with an increase in direction up to 90°. Thus, the variation in TT can be largely explained by changes in RT rather than those in MT.

Two-way ANOVA with distance and direction as factors (3×7 ANOVA) was conducted for TT. Although no significant interaction was found (*F* [12, 180] = 1.68, *p* = 0.074, *η*^*2*^ = 0.004), significant main effects were observed for distance (*F* [2, 30] = 332.56, *p* = 1.11×10^−16^, *η*^*2*^ = 0.53) and direction (*F* [6, 90] = 28.91, *p* = 1.11×10^−16^, *η*^*2*^ = 0.067). Multiple Bonferroni-corrected comparisons revealed significant differences between 22.5° and 67.5° for all three distances. Significant differences were also found between 45° and 90° and 67.5° and 135° for the 25 cm and 50 cm distances. At a distance of 75 cm, a significant difference was observed between 45° and 112°.

**Fig 5:**
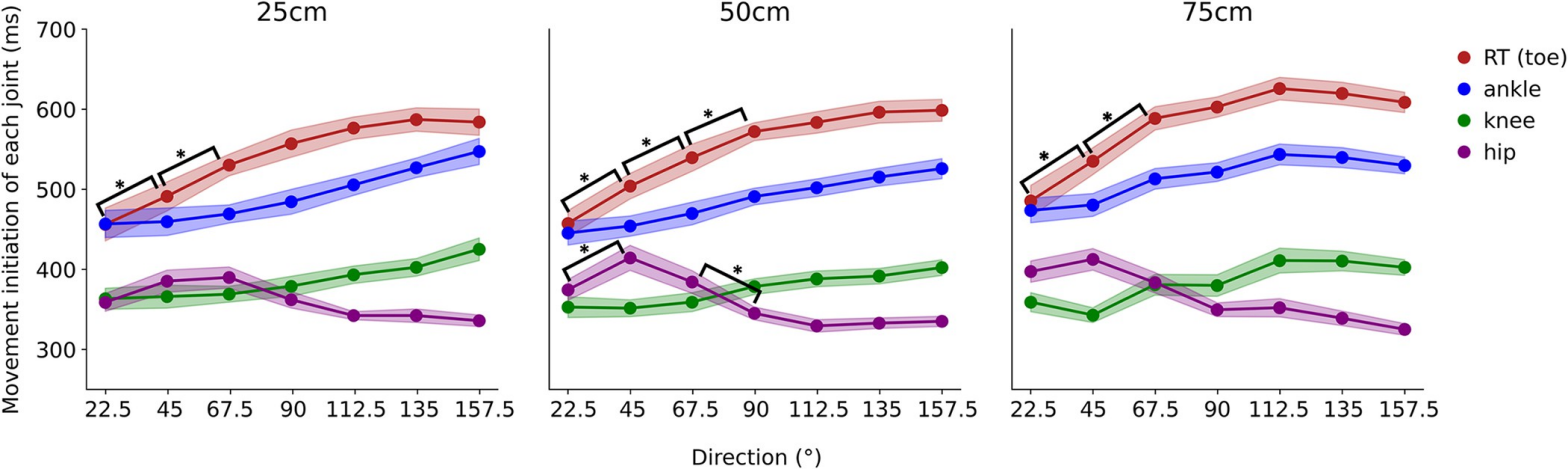
Movement initiation of each joint. The line color represents the joint, and the column the distance. The lightly colored bands indicate SE (Standard error). The significant difference between each joint at 0.5% level is shown.

We also conducted a 2-way ANOVA (3 × 7) with distance and direction as factors for RT. The results of multiple comparisons are shown in Fig 5 due to the configuration of the graph. Although no interaction was observed (*F* [12, 180] = 1.22, *p* = 0.27, *η*^*2*^ = 0.005), the main effects of distance (*F* [2, 30] = 16.42, *p* = 1.53×10^−5^, *η*^*2*^ = 0.048) and direction (*F* [6, 90] = 88.88, *p* = 1.11×10^−16^, *η*^*2*^ = 0.37) were observed. As a result of multiple Bonferroni-corrected comparisons, significant differences were observed between 22.5° and 45°, and between 45° and 67.5° at all three distances. In addition, a significant difference was observed between 67.5° and 90° at a distance of 50 cm.

For MT, a two-way ANOVA (3×7) was conducted with distance and direction as factors. The results showed no interaction effect (*F* [12, 180] = 1.18, *p* = 0.30, *η*^*2*^ = 0.002), but main effects were found for distance (*F* [2, 30] = 324.34, *p* = 1.11×10^−16^, *η*^*2*^ = 0.70) and direction (*F* [6, 90] = 18.42, *p* = 7.51×10^−14^, *η*^*2*^ = 0.017). Multiple Bonferroni-corrected comparisons revealed no significant differences between any direction at any of the three distances.

The average movement initiation values for each joint, which provide insight into the entire limb movement, are shown in Fig 5. RT and movement initiation of the ankle similarly increased with an increase in direction angle. However, this was not the case for the initiation of hip movement. Direction dependency of movement initiation of the hip was biphasic; movement initiation increased with an increase in direction angle up to approximately 45° and thereafter decreased with a further increase in direction angle. This trend was particularly prominent at a reaching distance of 50 cm (Fig 5. center panel).

To determine statistical significance, we conducted a three-factor ANOVA for movement initiation (3×7×4) using distance, direction, and joint (toe, ankle, knee, and hip). We found a significant two-way interaction effect (*F* [36, 540] = 5.69, *p* = 1.11×10^−16^, *η*^*2*^ = 0.003), a simple interaction effect between the joint and direction angle (*F* [18, 270] = 125.31, *p* = 1.11×10^−16^, *η*^*2*^ = 0.068), and a main joint effect (*F* [3, 45] = 391.40, *p* = 1.11×10^−16^, *η*^*2*^ = 0.64). Multiple comparisons of Bonferroni-corrected RT data revealed significant differences between 22.5° and 45° and between 45° and 67.5° at all distances. There was a significant difference in movement initiation of the hip duration between 22.5° and 45° and between 67.5° and 90° at 50 cm. There was a significant difference between RT and movement initiation of the ankle at all three distances and direction angles > 22.5°.

**Fig 6:**
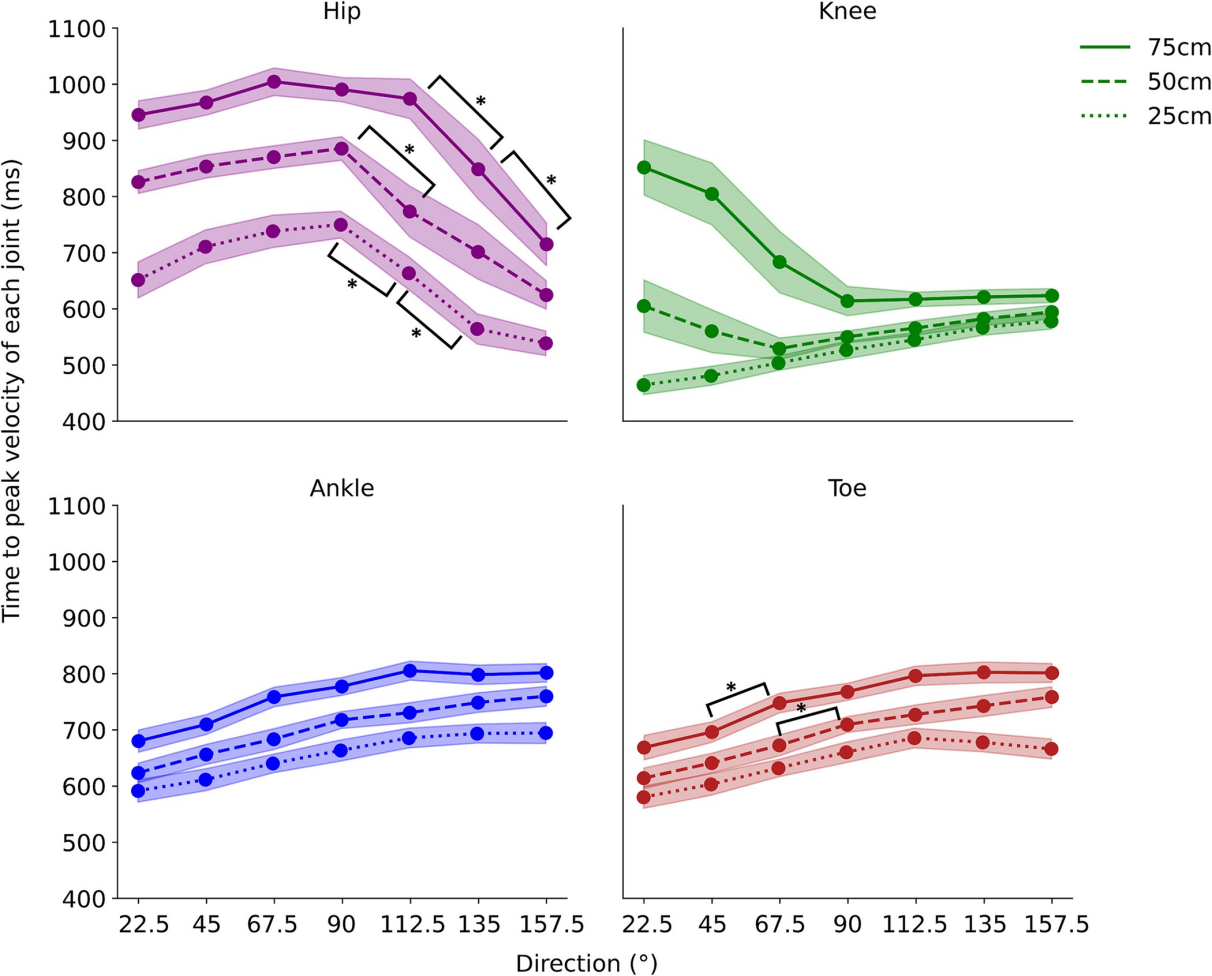
Time-to-peak velocity in the hip, knee, ankle, and toe. The lightly colored bands indicate the standard error (SE). Significant difference between each direction angle at 0.5% level is shown.

The average values of the time-to-peak velocity at each direction angle are shown in Fig 6. The time-to-peak velocity of the hip was higher than those of other joints and increased with the increase in direction angle up to 90°; thereafter, it decreased. The time-to-peak velocity of the knee linearly increased with an increase in the direction angle at a distance of 25 cm. In contrast, it decreased with an increase in the direction angle up to approximately 90° and increased afterwards. Thus, the time to peak velocity of the knee seems to converge to approximately the same value, irrespective of the distance, at higher direction angles (most contralateral side). The time-to-peak velocity of the ankle and toe largely increased with an increase in the direction angle.

A two-factor ANOVA of direction and distance was applied to the time-to-peak velocity (7×3 ANOVA was conducted four times). There was a significant simple interaction effect between distance and direction angle (*F* [12, 180] = 2.53, *p* = 0.004, *η*^*2*^ = 0.009) and a significant main effect of distance (*F* [2, 30] = 99.73, *p* = 5.57×10^−14^, *η*^*2*^ = 0.230) and direction angle (*F* [6, 90] = 71.95, *p* = 1.11×10^−16^, *η*^*2*^ = 0.228) on the time-to-peak velocity of the toe. Multiple comparisons of Bonferroni-corrected data revealed significant differences between 67.5° and 90° at 50 cm and between 45° and 67.5° at 75 cm.

No significant simple interaction effect was noted for the time-to-peak velocity of the ankle between distance and direction angle (*F* [12, 180] = 1.35, *p* = 0.19, *η*^*2*^ = 0.004); however, there was a significant main effect of distance (*F* [2, 30] = 81.22, *p* = 7.80×10^−13^, *η*^*2*^ = 0.226) and direction angle (*F* [6, 90] = 69.96, *p* = 1.11×10^−16^, *η*^*2*^ = 0.218). Multiple comparisons of the Bonferroni-corrected values revealed no significant difference between the adjacent direction angles.

We found a significant simple interaction effect between distance and direction angle (*F* [12, 180] = 15.39, *p* = 1.11×10^−16^, *η*^*2*^ = 0.150) and a significant main effect of distance (*F* [2, 30] = 59.05, *p* = 3.96×10^−11^, *η*^*2*^ = 0.245) and direction angle (*F* [6, 90] = 3.52, *p* = 0.004, *η*^*2*^ = 0.032) on the time-to-peak velocity of the knee. Subsequent multiple comparisons of Bonferroni-corrected data revealed no significant differences between adjacent direction angles.

A significant simple interaction effect between distance and direction angle was observed for the time-to-peak velocity of the hip (*F* [12, 180] = 1.19, *p* = 0.036, *η*^*2*^ = 0.012). Additionally, there was a significant main effect of distance (*F* (2, 30) = 111.25, *p* = 1.33×10^−14^, *η*^*2*^ = 0.342) and direction angle (*F* [6, 90] = 42.30, *p* = 1.11×10^−16^, *η*^*2*^ = 0.220). Multiple comparisons of Bonferroni-corrected data confirmed a significant difference between 90° and 112.5°at 25 cm and 50 cm, and between 112.5° and 135° at 75 cm.

Finally, it should be noted that there was no statistical evidence of progressive order effects which could have been due to adaptation or fatigue in any of the repeated-measures analyses.

## Discussion

Our results suggest that in lower-limb reaching movements, the time to reach the contralateral side was delayed, supporting our hypothesis. The reaching time distinctively increased with the increase in direction angle, indicating that movement to the contralateral side took more time than that required for movement to the ipsilateral side (Fig 4, left panel). This was largely due to the higher time to toe-off that could not be compensated for by the decrease in the time of toe movement with an increase in the direction angle (Fig 4, right panel). Previous studies have reported that the time when the heel lifts off the ground during contralateral movements is delayed, although the range of motion is limited [11]. Although our study’ findings are consistent with some findings of previous studies, we found that toe-off was delayed in a wider range of directions, particularly from 22.5° to 67.5°. The time to toe-off increased with an increase in the direction angle and predominated by the decrease in the time of toe movement on the contralateral side. This resulted in an overall increase in the reaching time with an increase in the direction angle (gray lines in Fig 4, right panel).

Based on these results, it is suggested that the reason for the slower arrival time of the target-directed movement on the opposite side is not the need to take a detour to reach the target, but rather because the time spent in preparing to lift the foot from the ground differs depending on the direction of movement. Previous research on walking has suggested that an insufficient step width increases the cost of movement because the swing leg takes a detour around the support leg [24]. However, in this study, the reaching movement was performed toward a target that spread fan-shaped from the manipulating foot; therefore, there was no difference in the distance traveled depending on the direction. Moreover, the support leg did not obstruct the distance from the starting position to the target, and there was no need for the swing leg to detour. Therefore, it is considered that the slower arrival time on the opposite side is not due to movement from the start to the target, but rather due to the preparation movement for lifting the foot from the ground being different, depending on the direction. This study provides evidence that factors affecting the preparation movement for lifting the foot from the ground can cause a delay in the arrival time to the opposite side.

As a factor that contributes to the delayed time to reach the opposite side, it became apparent that preparatory movements varied depending on the direction of movement. The movement initiation of each joint suggests that the movement differs between the ipsilateral and contralateral sides. The movement initiation of the toe and ankle was almost comparable for ipsilateral movement at 22.5°; however, the ankle was lower than the toe at the contralateral side beyond 45° (Fig 5). The hip was higher than the knee in the ipsilateral direction at < 67.5°, and this relationship was reversed for movements beyond 90°. This observation showed that the hip moved faster, followed by the knee, ankle, and toe, as the direction increased to the contralateral side. These results imply that the kicking motion on the contralateral side started from the body part closest to the trunk. This is especially true for movement in the farthest contralateral direction, where the action was primed by hip movement. The characteristics of the hip, such as instability of the center of mass during lateral reaching movements, are believed to necessitate the initial control of posture. Directional dependence of movements is thought to be related to gait initiation. Previous studies on gait initiation have reported that lateral displacement of the CoP increases during walking in the contralateral direction [11]. Our study results showed that movement to the contralateral side started from the body part closest to the trunk. From these results, it was suggested that there is a possibility that faster movement is needed to create a larger lateral CoP displacement. Thus, movement preparation before toe-off differs between movement toward the ipsilateral and contralateral sides, which may consequently modulate toe-off time.

Furthermore, in our results, tailored velocity strategies, adjusted according to each direction and distance, were observed in each joint (Fig 6). This implies that each joint changes its strategy of lifting the toe off the ground and the movement strategy during toe movement to reach the destination more quickly. Tailored velocity strategies for reaching movements has also been observed in upper-limb movements. Previous studies have reported that in horizontal reaching movements, the alignment of joints affects the time required to reach the maximum speed, which varies depending on the direction of the movement [1, 2, 25]. This is believed to be a result of adjusting the velocity strategies to maximize reaching efficiency for each movement direction. The present study found more complex relationships between the joints and distances because the movement of the lower limbs is necessary to support the body. As a general trend, it was confirmed that the time to reach the maximum speed became slower as the toe and ankle moved to the opposite side, while on the other hand, it was confirmed that the hip tended to become faster. The trend of the knee was biphasic, similar to that of the ankle and toe for close distances (25 cm), and similar to that of the hip for far distances (75 cm). This implies that the part of the limb farthest from the trunk, ankle, and toe moved consistently irrespective of the reaching distances and direction. The knee moved in a direction similar to that of the ankle or toe at close distances (25 cm) and similar to that of the hip at far distances (70 cm). The movement in the farthest contralateral direction was probably primed by the movement of the hip, followed by that of the knee, ankle, and toe. Therefore, it seems plausible that knee movement integrates the action of the limb parts closest to and farthest from the trunk, and plays a pivotal role in reaching the object on the contralateral side. We tested lower-limb reaching movements in this study and found that strategies for faster reaching may be complex, depending on distance, direction, and joint.

We observed two notable characteristics in the velocity profiles (Fig 3). First, the hip velocity profile was bimodal. This is because it includes two peaks: one as a preparatory movement until the toe leaves the ground and the other as a peak for the subsequent movement after the toe starts moving. This was particularly evident in the movements to the contralateral side. The first peak of the hip appeared before toe-off, which reached the contralateral side first and required movement transmission from the trunk to the toe. This bimodality was evident in our results that the hip was faster than the ankle on the contralateral side and reinforced the notion of movement transmission (Fig 6).

Second, the toe velocity profile differed to some extent from the bell shape described in previous studies. This is thought to be related to the addition of the function of supporting the body, which is specific to the lower limbs. Upper-limb reaching movements show a bell-shaped velocity profile [1, 26]. In our study, there was another shoulder peak just before the end of the movement, particularly at far distances and on the ipsilateral side. This may be due to the transmission of the second peaks of the knee or hip, which is a novel characteristic suggesting a connection between the joint parts during reaching movements. The characteristics discovered in this study are novel and unique to the lower limb.

## Limitations and future prospects

A limitation of this study was that the movement of the center of gravity could not be measured because data from the force plate were not obtained. When considering the movement of the lower limbs, it is necessary to consider the function of supporting the body, and the relationship between the manipulating and supporting legs is important. In this study, changes in the kinematics of each joint until the toe was lifted off the ground were confirmed. Furthermore, there is a potential limitation in precisely controlling the conditions as measurements for the weight distribution prior to the target onset were not obtained. Although instructions were given to avoid tilting the body, the potential variability in the tilt of the center of gravity before the presentation of the visual stimulus may influence the movement characteristics in different directions. Therefore, the findings should be interpreted with caution, acknowledging the possibility of unmeasured variations in the weight distribution before the visual stimulus. In the future, by incorporating data from the force plate, a deeper understanding of the movement of the lower limbs can be achieved, allowing for an examination of the influence of weight distribution prior to the target onset.

## Conclusion

The current study provides novel insights, as it shows that the reaching movements of the lower limb may change with direction and distance. Kinematic data demonstrated that movement preparation varies with direction, which changes the timing of toe-off. This characteristic is specific to the lower limbs. Overall, our findings highlight the involvement of joint transmission in the lower limb.
